# Construction of an empathy ability index system for clinical nurses using the ADDIE model: a Delphi study

**DOI:** 10.3389/fpsyg.2026.1868186

**Published:** 2026-09-28

**Authors:** Wanmin Huang, Hongjiao Xu, Guang Shi, Jinyu Chen, Limin Zhou

**Affiliations:** 1 Department of Oncology, The Affiliated Panyu Central Hospital, Guangzhou Medical University, Guangzhou, China; 2 Operation Room, The Affiliated Panyu Central Hospital, Guangzhou Medical University, Guangzhou, China; 3 Nursing Department, The Affiliated Panyu Central Hospital, Guangzhou Medical University, Guangzhou, China; 4 Human Resources Department, The Affiliated Panyu Central Hospital, Guangzhou Medical University, Guangzhou, China

**Keywords:** ADDIE model, Delphi study, empathy ability, nurse, nurse training

## Abstract

**Background:**

Empathy is closely related to the nursing process; it is a fundamental and essential quality required by nurses to cultivate a healthy nurse–patient relationship. However, no systematic or scientific model of empathy training is currently available for clinical nurses.

**Objective:**

We constructed a standardised training system for nurses’ empathy abilities based on the Analysis, Design, Development, Implementation, and Evaluation (ADDIE) Model, Davis’ multidimensional empathy theory, and empathy cycle theory.

**Design:**

An online two-round Delphi survey of empathy care experts was conducted.

**Methods:**

From January 2023 to July 2023, literature reviews, semi-structured interviews with regularly trained nurses, and group discussions were conducted to develop an indicator system for cultivating nurses’ empathy abilities. This involved 20 nursing experts from Beijing, Hubei, Jiangsu, Hainan, Shanxi, Guangdong, and Chongqing. The inclusion criteria for indicators were a mean score of ≥4 points and a coefficient of variation of <0.25. Combined with the research group’s in-depth analysis of the experts’ suggestions, a system for cultivating and practising nursing empathy abilities was formed.

**Results:**

After two Delphi rounds, the final index system included a three-part indicator system, with 14 first-level indicators, 24 s-level indicators, and 29 third-level indicators. The response rates for the two rounds were 100 and 85%, the proportions of experts providing suggestions were 60 and 47.06%, and the expert authority coefficient of the two rounds of consultation were 0.898 and 0.869, respectively.

**Conclusion:**

The educational pathway constructed to cultivate nurses’ empathy included ability training, training implementation, and training effect evaluation. This Delphi study established expert consensus and preliminary content validity for the indicator system. Future implementation studies with pre- and post-training assessments are needed to evaluate its feasibility and educational effectiveness in clinical nursing populations.

## Introduction

Empathy is the ability to understand a patient’s situation, feelings, and behaviour helpfully or therapeutically from the patient’s perspective (Kesbakhi and Rohani, 2020). It is an important part of medical humanities and a practical quality for nurses to cultivate a healthy nurse–patient relationship (Li et al., 2024). Empathy involves recognising another person’s inner experience and accurately identifying the core emotional components of that experience. Nursing empathy is characterised by the nurse’s ability to understand a patient’s feelings, experiences, and psychosocial status (Moudatsou et al., 2020). Additionally, the benefits of empathy are associated with patient outcomes, such as improved nurse–patient therapeutic communication, increased patient satisfaction, and improved person-centred care (Prado-Gascó et al., 2019; Brunero et al., 2022). What’s more, the nursing empathy can be developed through nurse education programme and training (Bowden et al., 2023). Therefore, an accurate and empathetic understanding of patients and effective emotional management skills are critical for achieving higher levels of patient well-being and comfort.

To address the need for a consensus on the effects of empathy training, it is important to note that there is substantial heterogeneity in studies using self-assessment and other assessment scales to evaluate empathy. Among these, the Hogan Empathy Scale, Interpersonal Reactivity Index (IRI), Empathy Construct Rating Scale, Reynolds Empathy Scale, Barrett-Lennard Relationship Inventory, and Jefferson Scale of Empathy (JSE) are frequently recommended for assessing self-perceived empathic ability or tracking its development over time, rather than serving as direct objective measures of the behavioral or clinical outcomes of such training (Giménez-Espert and Prado-Gascó, 2020).

Empathy training is important for nurses; the American College of Nursing Association emphasises its importance in nursing education (Wan et al., 2023). A previous study on empathy training primarily focused on evaluating the effects of a single training method (Kataoka et al., 2019) and neglected the development of a systematic training course. By this, we refer to a structured, longitudinal curriculum that moves beyond one-off interventions to include repeated exposure, progressive skill-building exercises, and continuous performance evaluation. Incorporating a curriculum that allows trainees to practice learned skills might be the most effective approach for enhancing empathy (Patel et al., 2019). Currently, empathic ability training mainly evaluates the effects of a single teaching method without a systematic and scientific training model (D'souza et al., 2020). However, another study has explored teaching empathy skills to intensive care unit (ICU) nurses (Mirzaei Maghsud et al., 2020). Therefore, developing courses or learning modules that incorporate empathy-based competencies among clinical nurses, grounded in a systematic model, is important.

Although empathy plays an important role in the clinical nurse–patient relationship, and teaching empathy impacts knowledge, perspective, and behaviour, only a few studies have focused on structured empathy training. Recent evidence demonstrates that empathy significantly predicts moral sensitivity in clinical nurses and is strongly correlated with ethical decision-making capacity (Barrett-Lennard, 1981), underscoring the need for systematic empathy education. Therefore, this study aimed to develop a preliminary indicator system for empathy training pathways for nurses.

## Materials and methods

### Theoretical framework

ADDIE is a systematic model that has been used as a teaching design framework. It can be an effective tool in nursing education, providing an established structure for developing educational programme (Kim et al., 2020). This model proposes a series of repeated steps to establish effective education and training, which are divided into five intertwined stages: analysis, design, development, implementation, and evaluation. By engaging learners as decision-makers and stimulating their interest in learning, the ADDIE model ensures a positive cycle of continuous improvement. This approach effectively changes the original monotonous training mode, improving nurses’ abilities (Ibrahim and Ibrahim, 2025). Another theoretical framework comprising Davis’ multidimensional empathy theory (Davis, 2013). Davis’ theory informed the content of empathy (cognitive, affective, behavioral), shaping the “Ability training” indicators. The onion model further guided the hierarchical structure of indicators (skills → attitudes → values), corresponding to the three indicator levels. The relationships among these frameworks are illustrated in Figure 1.

**Figure 1:**
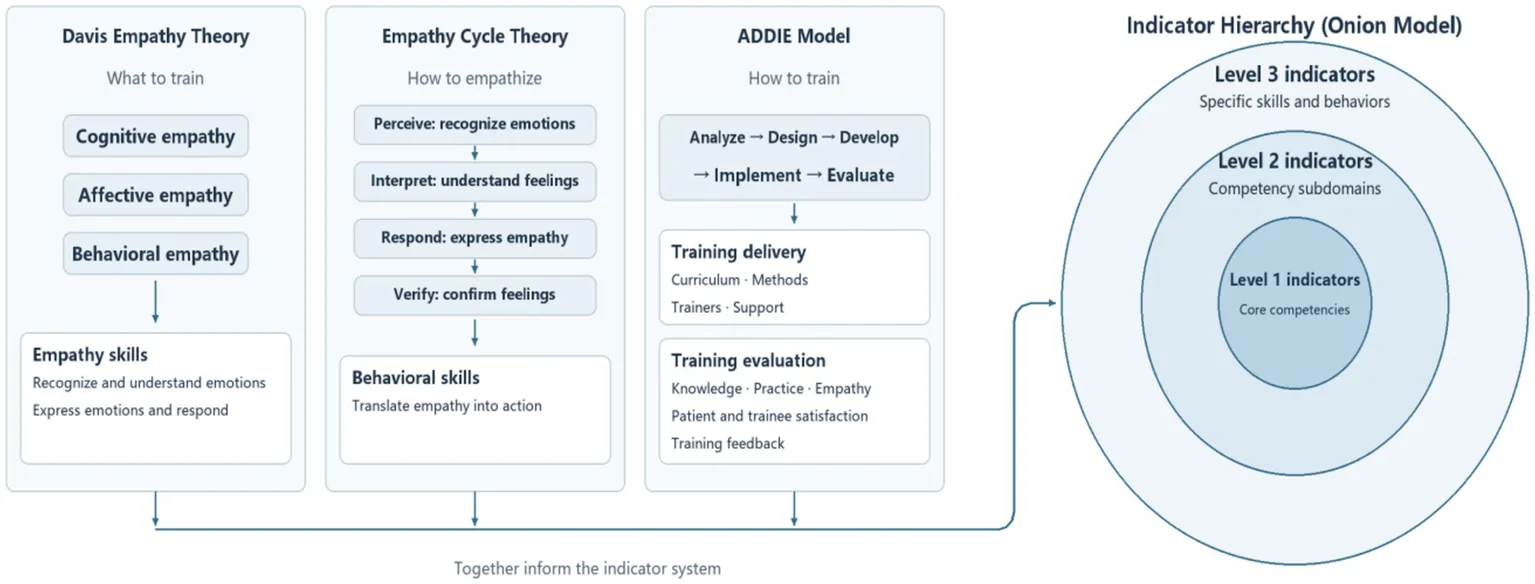
Theoretical framework for empathy training indicators.

### Expert recruitment and panel size

The number and qualifications of the experts recruited to serve on the panel were determined according to the methodology of the Delphi study. From January to July 2023, the research group used a purposive sampling strategy to recruit 20 experts in nursing education, humanistic nursing, nursing teaching management, and related fields for expert consultation. The inclusion criteria were: (1) educational background of bachelor’s degree or higher; (2) having certain research achievements in nursing education, humanistic nursing, or nursing teaching management and other related fields; and (3) having a junior title or higher. Experts who met the inclusion criteria were contacted for voluntary participation in this study. Those who agreed to participate were asked to confirm their consent by filling out an online consent form.

### Index system construction and questionnaire design

The Delphi method involves collecting data from a designated panel of experts, with 15–30 respondents per group, which is sufficient to achieve the study objectives if the experts are homogeneous (McPherson et al., 2018; Dijkstra et al., 2021). In this study, the Delphi method was used to reach a consensus on constructing empathy ability indicators for nurses in standardised training. The index system construction contained two stages:

1) Literature review and content analysis: A systematic literature search was conducted in PubMed, CINAHL, CNKI for publications from January 2000 to January 2023. The search combined terms related to: (a) population (nurs OR “nursing staff”); (b) concept (empath OR “empathic competence” OR “empathy ability”); and (c) context (“competency framework” OR “indicator system” OR “evaluation”). Eligibility criteria were: (a) studies published in English or Chinese; (b) focusing on nurses‘empathy or communication competencies; (c) proposing competency frameworks or evaluation tools; and (d) original research, reviews, or guidelines. Exclusion criteria were: (a) non-nurse populations; (b) empathy as a psychological trait without educational implications; and (c) conference abstracts or opinion pieces. Two researchers independently extracted meaning units—defined as the smallest discrete text segment conveying a distinct empathy-related idea—and coded them into preliminary categories. Inter-coder agreement was assessed, and discrepancies were resolved through consensus with a third researcher.
2) Group discussions and translation into candidate indicators: The research team—comprising 8 nursing educators, clinical nursing specialists, and medical education researchers—convened three discussion sessions to synthesize evidence from the literature review. The team: (a) compared literature-derived and interview-derived indicators; (b) identified overlaps and gaps; (c) consolidated redundant items and refined wording; (d) mapped all candidate indicators to Davis’ and ADDIE theoretical framework.
3) Delphi round: The Delphi technique was conducted using an online platform individually accessible by panelists. Two rounds of Delphi surveys were conducted to reach a consensus.

An expert consultation questionnaire was developed for the training of clinical nurses, consisting of three parts:

- Letter to experts: This section explained the significance, background, purpose, and specific details of this study.
- The three-level index evaluation table: This table for cultivating empathy ability in nurses included three parts: ability training, implementation, and effect evaluation. The importance of each indicator was evaluated using a 5-point Likert scale, from 5 (very important) to 1 (very unimportant). The questionnaire also included columns for “revisions” and “supplementary indicator recommendations.”
- Expert information: This section collected information from experts, including their ages, educational backgrounds, professional titles, research fields, and years of work experience.

### Data analysis

Statistical analyses were performed using China Excel 2019 and SPSS 26.0. The demographic characteristics of the sample are presented using descriptive statistics, including frequency and proportions. Analytical measurements are expressed as mean and standard deviation (SD), and the degree of coordination of expert opinion is expressed as the coefficient of variation (CV). The positive rate of experts was expressed as the questionnaire recovery rate (RR) and the percentage of recommended experts, with an RR > 70% indicating effective consultation (Mao et al., 2020). The degree of expert authority was expressed by the expert authority coefficient (Cr), calculated as Cr = (Ca + Cs)/2, where Ca represents the basis of the expert‘s judgment and Cs represents the expert’s familiarity with the research topic. A Cr of 0.70 is generally considered reliable, reflecting the high level of authority of experts in the field (Spranger et al., 2022).

Based on the scores of each indicator, elements were incorporated when the CV was <0.25 and the mean of the significance assignment values was >4.00 (Yang et al., 2023). If these requirements were not met simultaneously, the final selection of structural elements was determined based on expert modification recommendations and discussions by researchers.

Kendall’s coefficient of concordance (*W*) was used to confirm that overall panel agreement had improved or remained stable across rounds. Kendall’s *W* was computed for each round to evaluate the overall agreement among panelists across all items. A significant *p*-value (<0.05) indicated that the observed agreement was not due to chance. The strength of agreement was interpreted as weak (*W* = 0.1–0.3), moderate (*W* = 0.3–0.5), or strong (*W* > 0.5) (Jünger et al., 2017).

### Expert consultation

The first round focused on modifying the framework indicators, while the intent of the second round was to review the content within the fixed framework. Both rounds involved 10 domain experts with over 15 years of experience in clinical nursing practice, nursing education, and empathy-related training or research (Figure 2). Feedback was collected via structured questionnaires and consolidated through iterative consensus-building meetings. In the first round, 87% of proposed indicator modifications were accepted, primarily to enhance operational clarity and contextual relevance. The second round achieved 94% agreement on content validity, with minor refinements to terminology and measurement criteria. All revisions were documented and traceable to specific expert inputs.

**Figure 2:**
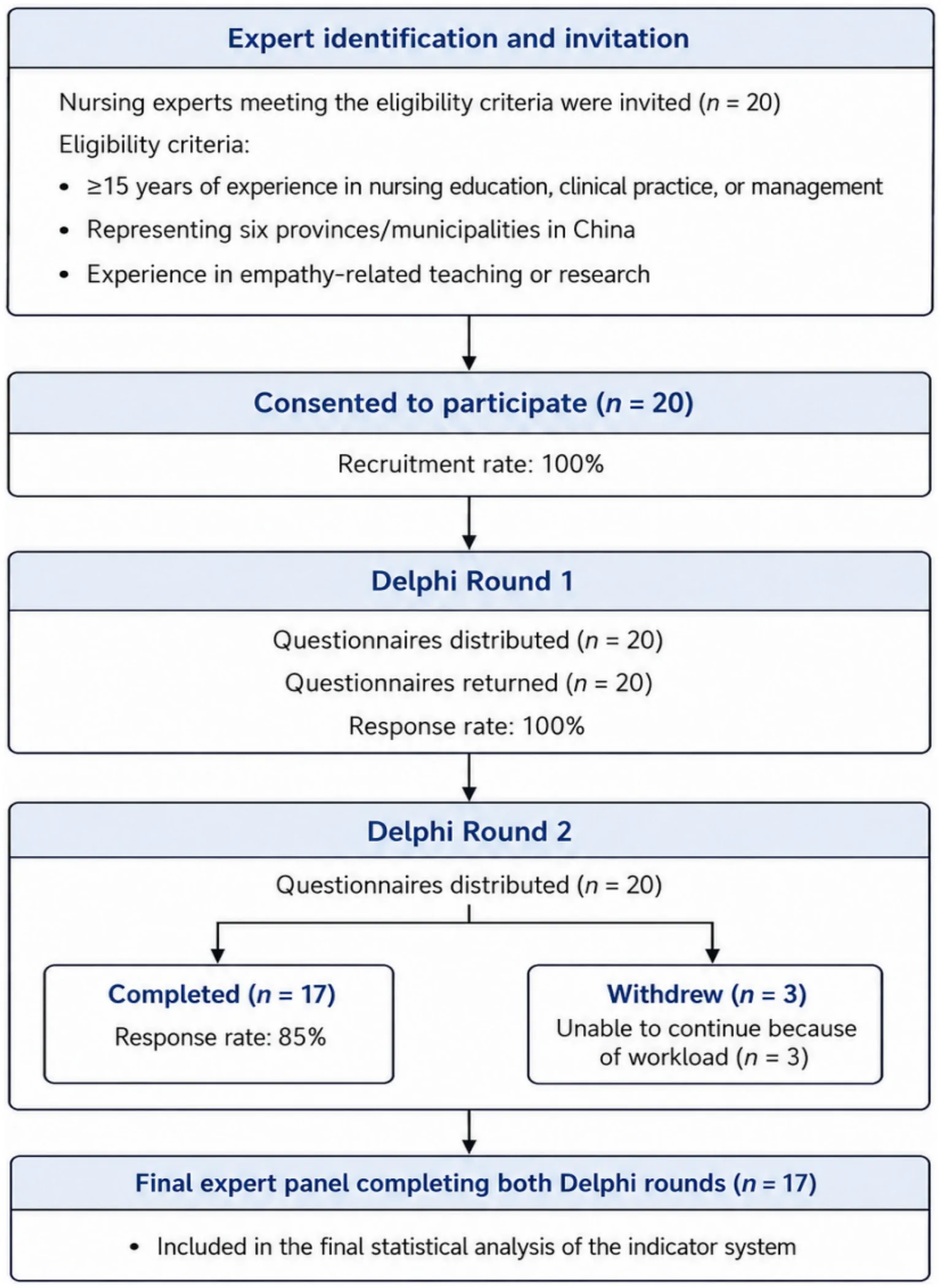
Expert identification and invitation.

### Ethical considerations

This study adhered to the principles of the World Medical Association Declaration of Helsinki and Chinese regulations on ethics. The Ethics Committee of the Guangzhou Panyu central Hospital for our study (Reference No.: PYRC-2024-114-01). Participants were informed of the purpose of the study, and informed consent was obtained. Anonymity and confidentiality of the information provided were ensured (World Medical Association, 2016).

## Results

### Demography and degree of authority of the experts

Demographic information of the experts was collected, involving 20 experts from six regions of China. All experts had over 15 years of experience in clinical nursing practice, nursing education, or nursing administration, with specific expertise in communication skills or empathy training. Their educational degrees were as follows: 3 had a doctorate (20.0%), 13 had a master’s degree (65.0%), and 3 had a bachelor’s degree (15.0%). Their mean total clinical experience was 27.90 ± 14.49 years (range: 15–54 years), with a mean age of 50.00 ± 11.31 years (range: 28–70 years). Their empathy-specific experience ranged from 3 to 54 years, reflecting diverse specialized engagement with empathy education. Regarding professional titles, 11 experts had senior titles (55.0%), 4 had intermediate titles (20.0%), and 5 had a junior title (25.0%) (Table 1).

**Table 1:** Information from the experts.

Characteristics	Round 1 (*n* = 20)	Percentage	Round 2 (*n* = 17)	Percentage
	*N* or (Mean ± SD)		*N* or (Mean ± SD)	
Age (years)	50.00 ± 11.31		46.71 ± 12.03	
Educational background				
Doctor	4	20.0%	3	17.6%
Master’s	13	65.0%	11	64.7%
Bachelor’s	3	15.0%	3	17.6%
Professional title				
Senior	11	55.0%	11	64.7%
Intermediate	4	20.0%	5	29.4%
Junior	5	25.0%	1	5.89%
Research field				
Nursing education	12	60.0%	9	52.9%
Nursing management	6	30.0%	7	41.2%
Nursing humanities	2	10.0%	1	5.9%
Working years	27.90 ± 14.49		24.00 ± 15.13	

### Activeness and authority coordination among experts

The expert positive coefficient, equivalent to the recovery rate of the expert questionnaire, indicates that a higher recovery rate corresponds to a higher expert positive coefficient. An expert positive coefficient exceeding 70% suggests that the questionnaire garnered active participation from experts (Jünger et al., 2017). In this study, 20 questionnaires were distributed during two rounds of expert correspondence. All experts participated in the first round. However, three experts did not return their questionnaires because of their busy schedules, resulting in 17 effective responses and an effective RR of 85%. The proportion of panelists providing suggestions varied between 60 and 47.06% across the two rounds. In the first round, 12 experts suggested modifications, while 8 experts suggested modifications in the second round.

### Validity and reliability of the Delphi indicators

#### Expert authority

The expert authority coefficient (Cr) was calculated using the formula Cr = (Ca + Cs)/2, where Ca represents the judgment basis (derived from theoretical analysis, practical experience, literature reference, and intuition) and Cs represents the familiarity with the research topic (rated on a 5-point scale from “not familiar” to “very familiar”). The mean Cr of the panel was 0.883 (range 0.869–0.898), exceeding the recommended threshold of 0.70, which confirms a high level of expert authority (Table 2).

**Table 2:** The degree of authority of the two rounds of expert letter consultation.

	Ca	Cs	Cr
First round (*n* = 20)	0.913	0.880	0.898
Second round (*n* = 17)	0.903	0.835	0.869

Cr, expert authority coefficient; Ca, judgment basis; Cs, familiarity.

#### Consensus reliability: coefficient of variation and Kendall’s *W*

In Round 1, the CV ranges were as follows: first-level indicators 0.08–0.11, second-level indicators 0.07–0.20, and third-level indicators 0.05–0.25. After modifications based on expert feedback, the second round yielded CV ranges of 0.08–0.10, 0.07–0.18, and 0.05–0.20, respectively. All CV values remained below 0.25, indicating acceptable consensus.

Kendall’s *W* increased from 0.195 (Round 1, *p* < 0.05) to 0.163 (Round 2,

*p* < 0.05), demonstrating a statistically significant but modest improvement in overall panel concordance. The detailed coordination coefficients are presented in Table 3.

**Table 3:** Harmonisation of expert opinions.

Item	The first round of expert correspondence		The second round of expert correspondence	
	Kendall’s *W*	*p*	Kendall’s *W*	*p*
Index	0.195	<0.001	0.163	<0.001

### Delphi round 1

A total of 96 opinions were provided in the first round of expert consultation based on the ADDIE model. The suggestions proposed by the experts were analyzed and the panellist made the following adjustments:

(1) Considering the structure of the first-level empathy training indicator

For the first-level indicators, the experts suggested revising the following revisions:

- Change “cognition of empathy” to “understanding of empathy”
- Modify “curriculum setting” to “total credit hours: 8 credit hours”
- Add two new second-level indicators in the training implementation part: “empathy case analysis” and “narrative education”
- Include “practice assessment” in the training effect evaluation

For the second-level indicators, the experts suggested deleting the following two indicators: “interpersonal relationship” and “peer evaluation.”

(2) Considering the structure of the second-level empathy training indicator

The experts suggested:

- Merging “emotional regulation” into “empathy response”
- Revising “carry out humanistic and empathy nursing practice for more than 1 year” to “3 years”
- Changing “self-evaluation” to “empathy ability evaluation”

(3) Considering the structure of the third-level empathy training indicators

For the third-level indicators, experts recommended deleting the following details:

- “Personalized patient care plan based on patient mood”
- “Skills and methods to identify bad emotional reactions”
- “Reflect on the life experience of different groups from themselves, understand the situation of others”
- “Master empathy”

Experts suggested revising three third-level indicators as follows:

- Modify the “concept, significance, and function of empathetic nursing” to “meaning and function of empathy practice in nursing”
- Change “guided listening” to “guided” and revise “master” to “guided type”

In addition, the mean importance scores of each indicator from the first round of expert consultation ranged from 4 to 5, with the CV values ranging from 0.06 to 0.23. Therefore, these indicators were not removed from the statistical analysis. Ultimately, 14 first-level, 24 s-level, and 29 third-level indicators were selected to form the questionnaire for the second round of Delphi.

### Delphi round 2

(1) Considering the structure of the empathy training method

In the second round of expert consultation, 10 opinions were presented. The experts suggested increasing the course duration. After reviewing the studies and experts’ discussions, there was an agreement to extend the duration to “8–16 credit hours.” The Balint group approach was retained, as it does not require a trainer with a psychological background to implement within the training context. “Narrative education” was also retained, as the expert panel reached a consensus on its value for enhancing reflective empathy skills. The suggestion to add video training was discussed but was not adopted, as the panel considered it less essential than the existing training methods.

(2) Considering the structure of the empathy training evaluation

The experts suggested that the training effect evaluation should follow the order of theory, practice, empathy ability evaluation, patient satisfaction evaluation, training satisfaction evaluation, and training feedback evaluation. Additionally, it was recommended to increase the emphasis on “affective cognition” by adding “the importance of affective cognition”; this suggestion was adopted.

(3) Final indicators

In the second round of Delphi consultation, the importance scores for each indicator ranged from 4 to 5, while the CV values ranged from 0.049 to 0.161—all below the predefined consensus threshold of 0.25 (see Table 4). The third round was not considered essential for the following reasons: (1) all indicators met the *a priori* consensus criteria (mean > 4.00 and CV < 0.25) in Round 2; (2) the number of expert opinions dropped from 96 in Round 1 to 10 in Round 2 (an 89.6% reduction), indicating that consensus had substantially stabilized; (3) Kendall’s *W* was statistically significant in both rounds (Round 1: *W* = 0.195, *χ*^2^ = 272.348, *p* < 0.001; Round 2: *W* = 0.163, *χ*^2^ = 185.329, *p* < 0.001), confirming that the observed agreement among experts was unlikely to be due to chance; and (4) the revisions made after Round 2 were minimal (course duration extension, evaluation sequence reordering, and emphasis on affective cognition)—no indicators were added, deleted, or merged. Thus, termination after two rounds was justified, consistent with established Delphi methodological practices (Hasson et al., 2000). Figure 3 for the complete revision process across all stages. Ultimately, 14 first-level, 24 s-level, and 29 third-level indicators were selected for the structure of the second Delphi round.

**Table 4:** Evaluation index system of the core competence of empathy for training nurses.

Indicator system	First-level indicators	Second-level indicators	Third-level indicators	Round 1			Round 2		
				Mean ± SD	CV	Weight	Mean ± SD	CV	Weight
Ability training									
	1. Identify emotions			4.85 ± 0.366	0.076	0.012	4.647 ± 0.493	0.106	0.013
		1.1 Interpretation of emotions		4.65 ± 0.933	0.201	0.021	4.706 ± 0.470	0.100	0.014
			1.1.1 The concept and classification of emotions	4.600 ± 0.598	0.130	0.013	4.353 ± 0.606	0.139	0.012
			1.1.2 Skills and methods to identify their emotions	4.80 ± 0.410	0.085	0.012	4.647 ± 0.493	0.106	0.014
		1.2. Emotional cognition		4.80 ± 0.410	0.085	0.012	4.706 ± 0.470	0.100	0.014
			1.2.1 The concept, content, and meaning of emotional cognition	–	–	–	4.412 ± 0.618	0.140	0.012
			1.2.2 Classification of the affective responses	4.30 ± 1.031	0.240	0.023	4.353 ± 0.702	0.161	0.012
	2. Emotional recognition			4.85 ± 0.489	0.101	0.011	4.706 ± 0.470	0.100	0.014
		2.1. Emotional empathy		–	–	–	4.647 ± 0.493	0.106	0.014
			2.1.1 The concept, purpose, and function of empathy	4.75 ± 0.550	0.116	0.014	4.412 ± 0.618	0.140	0.012
			2.1.2 The significance and role of emotional empathy practice in nursing	–	–	–	4.588 ± 0.507	0.111	0.014
			2.1.3 Master empathy for others through the cognitive chain of emotional reactions	4.80 ± 0.410	0.085	0.012	4.765 ± 0.437	0.092	0.015
		2.2. Cognitive empathy		–	–	–	4.706 ± 0.470	0.100	0.014
			2.2.1 The concept, purpose, and role of cognitive empathy	–	–	–	4.471 ± 0.624	0.140	0.012
			2.2.2 The significance and function of cognitive empathy practice in nursing practice	–	–	–	4.529 ± 0.514	0.114	0.014
			2.2.3 Mobilise the cognitive chain to master the understanding of others' behaviour	–	–	–	4.765 ± 0.437	0.092	0.015
	3. Empathetic response			4.90 ± 0.308	0.063	0.012	4.765 ± 0.437	0.092	0.015
		3.1. Emotional response		4.80 ± 0.410	0.085	0.012	4.471 ± 0.514	0.115	0.013
			3.1.1 Can empathically respond to others' emotions, situations, and thoughts	4.85 ± 0.366	0.076	0.012	4.471 ± 0.717	0.160	0.017
			3.1.2 Correcting for cognitive bias to exercise personal empathy for others	4.45 ± 1.050	0.236	0.022	4.471 ± 0.624	0.140	0.018
			3.1.3 Combine your own opinions and the experiences of others	4.60 ± 0.940	0.204	0.022	4.647 ± 0.606	0.130	0.018
		3.2. Transpositional consideration		4.90 ± 0.308	0.063	0.012	4.706 ± 0.588	0.125	0.018
			3.2.1 The concept, purpose, and function of empathy	4.80 ± 0.410	0.085	0.012	4.353 ± 0.702	0.161	0.016
			3.2.2 Methods and techniques of empathy	4.85 ± 0.366	0.076	0.012	4.706 ± 0.588	0.125	0.018
		3.3. Guided listening		4.85 ± 0.366	0.076	0.013	4.706 ± 0.470	0.100	0.014
			3.3.1 The concept and function of guided listening	–	–	–	4.353 ± 0.606	0.139	0.012
			3.3.2 The importance and significance of guided listening	–	–	–	4.529 ± 0.514	0.114	0.013
			3.3.3 Guided listening methods, skills, and precautions	–	–	–	4.706 ± 0.470	0.100	0.014
		3.4. Emotional regulation		–	–	–	4.824 ± 0.393	0.081	0.015
			3.4.1 Concept and content of emotion management	–	–	–	4.412 ± 0.618	0.140	0.013
			3.4.2 Methods and skills of emotion management	–	–	–	4.647 ± 0.493	0.106	0.014
			3.4.3 Can sense the feelings of others and regulate their own emotions	–	–	–	4.529 ± 0.624	0.138	0.017
	4. Emotional expression			4.850 ± 0.366	0.076	0.012	4.765 ± 0.437	0.092	0.015
		4.1. Verbal communication		4.850 ± 0.366	0.076	0.012	4.824 ± 0.529	0.110	0.018
			4.1.1 The definition, meaning, characteristics, and type of language communication	–	–	–	4.294 ± 0.588	0.137	0.012
			4.1.2 The way of language communication and its influencing factors	–	–	–	4.588 ± 0.507	0.111	0.014
			4.1.3 Methods and steps of effective communication	4.70 ± 0.571	0.122	0.013	4.706 ± 0.470	0.100	0.014
			4.1.4 Language communication skills using empathic communication in interactions with patients	4.75 ± 0.444	0.094	0.012	4.765 ± 0.437	0.092	0.015
		4.2. Non-verbal communication		4.750 ± 0.444	0.094	0.012	4.765 ± 0.562	0.118	0.018
			4.2.1 Definition and type of non-verbal communication	4.350 ± 0.745	0.171	0.013	4.353 ± 0.606	0.139	0.012
			4.2.2 The way of non-verbal communication and its influencing factors	4.500 ± 0.607	0.135	0.014	4.471 ± 0.514	0.115	0.013
			4.2.3 Methods and Steps for Effective non-verbal Communication	4.700 ± 0.571	0.122	0.012	4.706 ± 0.470	0.100	0.015
			4.2.4 Use and well-bearing skills of empathic noncommunication in interaction with patients	4.800 ± 0.523	0.109	0.012	4.647 ± 0.493	0.106	0.014
Training implementation									
	1. Course setting: total credit hours: 8-16 credit hours			–	–	–	4.765 ± 0.437	0.092	0.015
	2. Training form			4.900 ± 0.308	0.063	0.012	4.824 ± 0.393	0.081	0.015
		2.1. Theoretical teaching		4.650 ± 0.587	0.126	0.013	4.529 ± 0.514	0.114	0.013
		2.2. Role Playing		4.800 ± 0.410	0.085	0.013	4.824 ± 0.393	0.081	0.015
		2.3. Balint Group		4.700 ± 0.571	0.122	0.013	4.647 ± 0.606	0.130	0.014
		2.4. Scenario simulation		4.800 ± 0.410	0.085	0.012	4.647 ± 0.606	0.130	0.019
		2.5. Panel discussion		4.650 ± 0.745	0.160	0.010	4.412 ± 0.618	0.140	0.017
		2.6. Empathy case sharing		–	–	–	4.647 ± 0.606	0.130	0.018
	3. Training support			4.850 ± 0.489	0.101	0.013	4.941 ± 0.243	0.049	0.016
		3.1. Training Manual		4.600 ± 0.681	0.148	0.013	4.471 ± 0.717	0.160	0.012
		3.2. WeChat support team (course tracking and Q & A)		4.650 ± 0.489	0.105	0.013	4.412 ± 0.507	0.115	0.013
	4. Training teachers			4.900 ± 0.308	0.063	0.012	4.941 ± 0.243	0.049	0.016
		4.1. Engaged in psychology/clinical nursing/nursing education for over 5 years		4.750 ± 0.550	0.116	0.013	4.882 ± 0.332	0.068	0.016
		4.2. Training in humanistic and empathic care		4.850 ± 0.366	0.076	0.013	4.882 ± 0.332	0.068	0.016
		4.3. Have rich experience in nursing practice		4.900 ± 0.308	0.063	0.012	4.882 ± 0.332	0.068	0.016
		4.4. Familiar with empathic nursing theory and related application and practice		4.850 ± 0.366	0.076	0.012	4.824 ± 0.393	0.081	0.015
		4.5. Humanistic and empathy nursing practice for more than 3 years		–	–	–	4.647 ± 0.493	0.106	0.014
Training effect evaluation									
	1. Training theory test			4.300 ± 1.174	0.273	0.012	4.412 ± 0.507	0.115	0.013
	2. Practice assessment			–	–	–	4.824 ± 0.393	0.081	0.015
	3. Empathy ability evaluation			–	–	–	4.882±0 ± .332	0.068	0.016
	4. Patient satisfaction evaluation			4.400 ± 1.231	0.280	0.010	4.765 ± 0.437	0.092	0.017
	5. Training course satisfaction evaluation			4.400 ± 1.231	0.280	0.012	4.647 ± 0.493	0.106	0.014
	6. Training feedback			4.450 ± 1.146	0.258	0.011	4.765 ± 0.437	0.092	0.015

**Figure 3:**
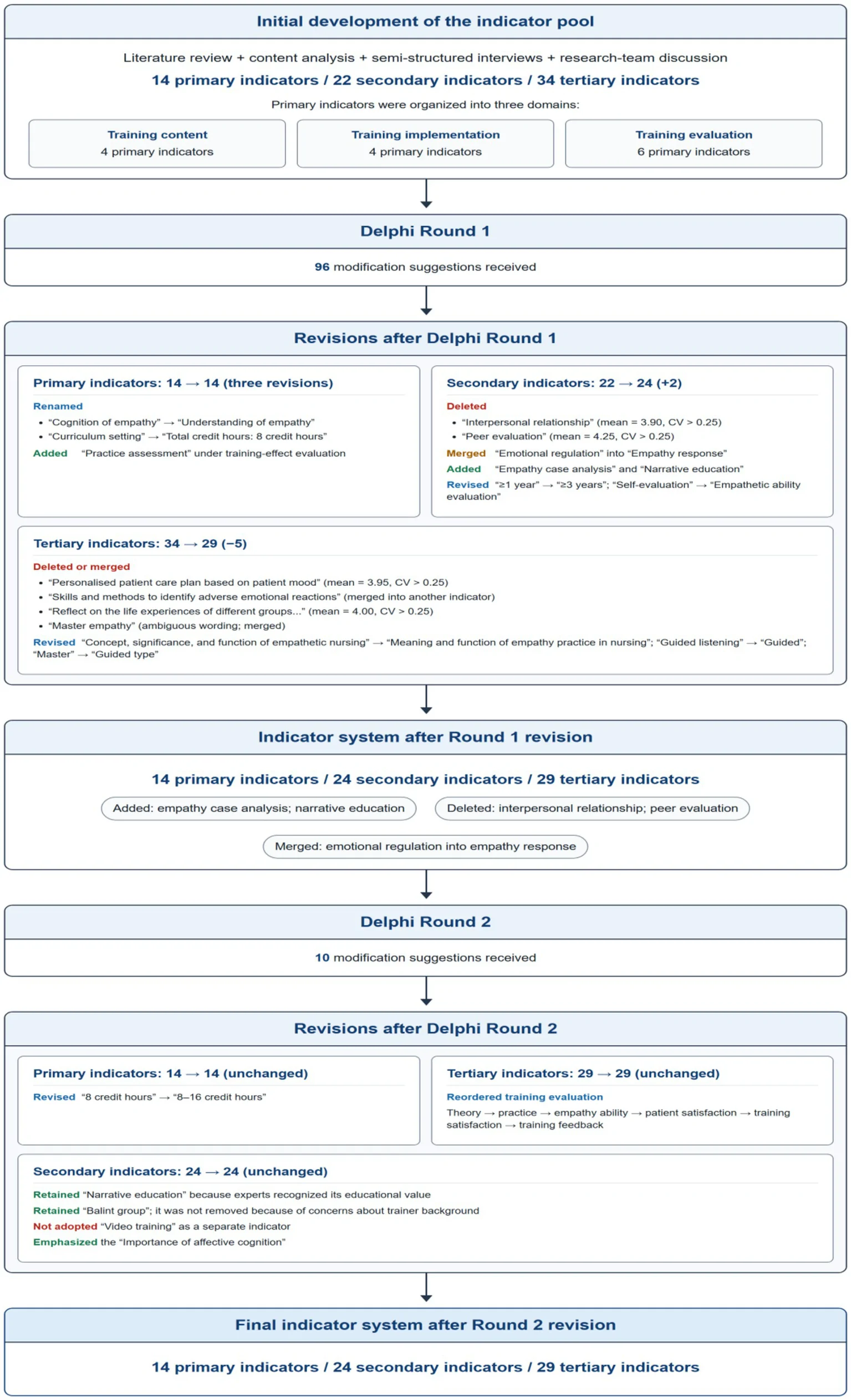
Evolution of the indicator system across Delphi rounds.

## Discussion

Based on the existing literature and two rounds of the Delphi survey, this study initially constructed an index system framework for evaluating nurses’ empathy abilities. This framework includes ability training, training implementation, and training effect evaluation, demonstrating reliability and scientific significance. It is suitable for clinical nurses’ empathy competency training and contributes to the development of a systematic and targeted training programme. To exhibit empathy is to also demonstrate caring behaviour toward patients, which can influence patients’ well-being and health outcomes (Atta et al., 2024). Therefore, demonstrating empathy toward patients can take the form of more personalized care and health support by nurses, ultimately benefiting patients’ health outcomes and improving their satisfaction.

Previous research has demonstrated that the ADDIE model is effective in clinical ethics education (Kim et al., 2020). This study highlights an empathy index system based on the ADDIE model. It suggests that while training theories often focus on training content and design as sources of effectiveness, they sometimes underestimate the role of the trainer (Glerum et al., 2021). As a new teaching theory, the ADDIE model has been used in cultural care curriculum design (Farsangi et al., 2023). Experts believe that trainers significantly impact trainees’ reactions and course evaluations, occasionally more than the course content. Therefore, trainers should be well-versed in the training content, possess a solid theoretical foundation, have extensive practical experience, and exhibit an authoritative teaching style to improve trainees’ self-efficacy and facilitate skill development (Ahn et al., 2023). The ADDIE model provides a systematic process that avoids training one-sidedness, ensuring comprehensiveness and consistency (Hakim et al., 2024). It also emphasises trainees’ needs and goals, allowing flexibility to adjust the training programme at each model stage, thereby aligning the training content with actual needs (Tsai and Chou, 2023).

A closer examination of the expert consensus reveals that empathy training cannot be treated as a monolithic construct; rather, its multidimensional nature demands differentiated training targets. Within the “ability training” dimension, the framework distinguishes between affective empathy and cognitive empathy. Affective empathy involves the automatic sharing of others’ emotional states and can be enhanced through experiential exercises, whereas cognitive empathy requires perspective-taking and mentalizing about others’ intentions, relying more on executive control and reasoning (Öztürk and Kaçan, 2022). This dissociation implies that training must target both “feeling with” and “understanding about” the patient. Furthermore, the emphasis on transpositional consideration—actively adopting the patient’s viewpoint—suggests that experts prioritise behavioral translation of empathic understanding over passive emotional reception (Ratka, 2018). Notably, the inclusion of emotional regulation as a core indicator reflects expert recognition that modulating one’s own emotions to prevent empathy fatigue is integral to competence, with particular relevance for nurses in high-stress clinical settings.

Experts reached a consensus that empathy training for clinical nurses should prioritize core skills—including guided listening, verbal communication, and non-verbal communication—rather than merely making empathic statements (Lee and Ihm, 2021). They also emphasized that empathy practice requires cross-cultural communication skills, as cultural backgrounds influence empathy expression (Molewyk Doornbos et al., 2014). Regarding training methods, role-playing and scenario simulation were endorsed as the most commonly used approaches (Fioravanti et al., 2018), while the Balint group was considered particularly conducive to empathy capacity training by providing opportunities to explore emotions and nurse–patient interaction dynamics (Douglas and Feeney, 2017). Notably, the optimal duration of Balint group interventions should be considered when developing training plans (Gong et al., 2024). This pattern of endorsed methods reflects a clear expert preference for experiential over didactic approaches, further corroborated by the retention of empathy case sharing. Such methodological differentiation underscores the importance of diverse, context-sensitive training modalities that address both the cognitive and affective dimensions of empathy.

When interpreting the expert consensus on training implementation, a clear preference for experiential over didactic methods emerges. Scenario simulation and role-playing were prioritized over purely theoretical teaching, while the retention of empathy case sharing further corroborates the training philosophy of “learning empathy in relationships” (Zhou et al., 2025). The inclusion of the Balint group, though complementing rather than substituting simulation-based approaches, offers unique value by providing a safe space for nurses to explore emotional dynamics within nurse–patient interactions. This methodological differentiation underscores the importance of diverse, context-sensitive training modalities that address both the cognitive and affective dimensions of empathy.

While these face-to-face methods are valued for their interactive intensity, the expert panel also recognised the need for post-class support to sustain learning, leading to the endorsement of supplementary digital tools. Experts also agreed that the multidimensional nature of empathy necessitates flexible training methods. While face-to-face learning is preferred for empathy skill development due to its interactive nature (Asghar et al., 2022), its limitation—lack of post-class instructor support—can be addressed through digital tools. The retained indicator “We Chat support team” (3.2) reflects expert consensus that digital platforms can provide supplementary after-class Q&A and course tracking, complementing rather than replacing in-person training (Tudor Car et al., 2019).

Regarding training evaluation, the multidimensional nature of empathy complicates the assessment of empathy-enhancing strategies. Among validated tools, the Jefferson Scale of Empathy (JSE) and Interpersonal Reactivity Index (IRI) are the most widely used. The JSE, which reflects the significance of empathy in healthcare settings, has been psycho-metrically validated specifically for nursing populations, demonstrating good reliability and validity (Hojat et al., 2018). To capture training effects, we recommend administering the JSE as a pre- and post-training assessment to measure changes in empathy levels. Such assessments also reveal individual learning differences, enabling trainers to develop personalized educational strategies within the ADDIE framework (Dong et al., 2025). Furthermore, experts emphasised that continuous interaction with trainees is essential for sustaining clinical behaviour change; accordingly, they endorsed increased practice assessment to maintain the continuity of empathy training.

## Study limitations and strengths

Several limitations should be acknowledged. First, our Delphi panel comprised 20 experts in Round 1, with 17 completing both rounds. While acceptable (10–30 participants), attrition and purposive sampling via professional networks may have introduced bias. Second, the panel predominantly comprised nursing educators/administrators with limited front-line clinician representation, and patient perspectives were excluded. Third, all experts were from China, limiting generalization without cross-cultural adaptation. Fourth, and most importantly, this study established expert consensus and content validity-not educational effectiveness or clinical feasibility. The system remains untested in practice; its construct validity, reliability, responsiveness, and utility require empirical evaluation. Expert consensus does not equate to demonstrated effectiveness. This system is a preliminary framework requiring further validation before clinical implementation.

## Conclusion

This Delphi study is an important and feasible part of constructing a standardised training system for nurses’ empathy abilities. Guided by Davis’ multidimensional empathy theory—which conceptualizes empathy as comprising both cognitive and affective dimensions—and operationalized within the iterative ADDIE instructional framework, the study reached a consensus on a set of core empathy skills. However, this system is a preliminary framework requiring further empirical validation—including feasibility testing, psychometric evaluation, and effectiveness studies—before it can be considered ready for clinical implementation. Therefore, the system should not be described as a “validated index system” at this stage, but rather as a theoretically grounded and expert-validated framework requiring further empirical evaluation.

## Data Availability

The raw data supporting the conclusions of this article will be made available by the authors, without undue reservation.
